# Prophylactic negative-pressure wound therapy after ileostomy reversal for the prevention of wound healing complications in colorectal cancer patients: a randomized controlled trial

**DOI:** 10.1007/s10151-020-02372-w

**Published:** 2020-11-07

**Authors:** M. Wierdak, M. Pisarska-Adamczyk, M. Wysocki, P. Major, K. Kołodziejska, M. Nowakowski, T. Vongsurbchart, M. Pędziwiatr

**Affiliations:** 2nd Department of General Surgery, Jagiellonian University Medical College, Jakubowskiego 2 Str., 30-688 Krakow, Poland

**Keywords:** Prophylactic negative pressure wound therapy, Ileostomy closure, Prevention of wound-related complications, Colorectal cancer operation, Randomized controlled trial

## Abstract

**Background:**

The aim of this study was to assess the usefulness of protective negative-pressure wound therapy (NPWT) in the reduction of wound healing complications (WHC) and surgical site infections (SSI) after diverting ileostomy closure in patients who underwent surgery for colorectal cancer.

**Methods:**

In this prospective randomized clinical trial in a tertiary academic surgical center, patients who had colorectal cancer surgery with protective loop ileostomy and were scheduled to undergo ileostomy closure with primary wound closure from January 2016 to December 2018 were randomized to be treated with or without NPWT. The primary endpoint was the incidence of WHC. Secondary endpoints were incidence of SSI, length of postoperative hospital stay (LOS), and length of complete wound healing (CWH) time.

**Results:**

We enrolled 35 patients NPWT (24 males [68.6%]; mean age 61.6 ± 11.3 years), with NPWT and 36 patients (20 males [55.6%]; mean age 62.4 ± 11.3 years) with only primary wound closure (control group). WHC was observed in 11 patients (30.6%) in the control group and 3 (8.57%) in the NPWT group (*p* = 0.020). Patients in the NPWT group had a significantly lower incidence of SSI (2 [5.71%] vs. 8 [22.2%] in the control group; *p* = 0.046) as well as significantly shorter median CWH (7 [7–7] days vs. 7 [7–15.5] days, *p* = 0.030). There was no difference in median LOS between groups (3 [2.5–5] days in the control group vs. 4 [2–4] days in the NPWT group; *p* = 0.072).

**Conclusions:**

Prophylactic postoperative NPWT after diverting ileostomy closure in colorectal cancer patients reduces the incidence of WRC and SSI.

**Clinical trial registration:**

clinicaltrials.gov (NCT04088162).

## Introduction

Surgeries for colorectal cancer, especially procedures involving the lower rectum, are associated with a very high percentage of complications [1–3]. Despite the current recomsmendation for primary anastomosis after a resection procedure, such treatment is associated with a significant risk of anastomotic leak (up to 20% = [4, 5]). One way to reduce the risk of leak is to use a diverting ileostomy [6, 7]. Although this is currently considered a standard treatment, especially in the group of patients undergoing neoadjuvant radiotherapy or chemoradiotherapy, the technique still has its drawbacks, such as the necessity to perform an additional surgical operation for ileostomy closure, which has a high risk of wound healing complications, particularly surgical site infections (SSI) [8, 9]. To reduce the frequency of these complications, novel guidelines published in 2017 recommend using the purse-string suture technique [9–11]. However, this has been associated with as much as a fivefold increase in healing time, as well as less desirable cosmetic effects, in patients without SSI [12, 13]. Negative-pressure wound therapy (NPWT) is currently a widely used method of treatment in various types of infectious complications, potentially providing the opportunity to prevent infectious complications after surgery [14, 15] by combining the benefits of both postoperative wound closure techniques: reduced healing time, compared to primary closure, and reduced risk of infectious complications, compared to purse-string techniques.

The aim of this randomized controlled trial was to assess the usefulness of postoperative NPWT in the reduction of postoperative wound-healing complications (WHC) and SSI after diverting ileostomy closure in patients who underwent colorectal resection for cancer.

## Materials and methods

### Patients

A randomized controlled trial was conducted between January 2016 and December 2018 in a tertiary referral center, University Hospital (Krakow, Poland). The trial was registered at clinicaltrials.gov (NCT04088162) after approval of the protocol by ethics committee of Jagiellonian University Medical College (#1072.6219.263.2019). The trial was designed as a single-center, randomized controlled, superiority trial with two parallel intervention arms.

### Participants

Patients aged ≥ 18 years with a history of surgery for colorectal cancer, including formation of the protective ileostomy, who were scheduled to undergo ileostomy closure as an elective procedure, were randomly divided into two groups: Group 1 to undergo postoperative NPWT and Group 2, a control group to undergo customary care (without postoperative NPWT). Patients were enrolled after providing informed consent on admission. Exclusion criteria were emergency/urgent operation, active infection, operations other than ileostomy closure, or parastomal hernioplasty. Patients who required a second operation or transfer to the intensive care unit or other hospital wards because of noninfectious complications within the first week after surgery were also excluded from the analysis.

### Randomization

The 1:1 randomization with concealment was achieved using a random number generator (even/odd) [16]. Until the end of the operation, patients did not know to which group they were assigned. The randomization process and assignment of the patients to the groups were performed by a trial researcher who was not directly involved in the operation or postoperative care of the patient. Operating surgeons were also blinded to the randomization. The NPWT dressing was set up at the end of the operation in sterile conditions in the operating room by one and the same person (the designated surgeon, a member of the research team) not directly involved in the operation or postoperative patient care.

### Sample size calculation

Our previous observations found the primary endpoint incidence in our population to be 33%, which was consistent with the data available in the literature [8, 9]. Additionally, a previous pilot study demonstrated that NPWT had decreased the incidence of WHC by 70–85% [17]. To demonstrate that NPWT decreased WHC by 80%, a total sample size of 70 subjects was needed for an alpha of 0.05 and 80% power. Thus, with expectations of omissions, we sought a total sample size of 38 patients in each arm.

### Procedures

Patients’ demographics, possible SSI risk factors, including age, sex, body mass index (BMI), active smoking, preoperative immunosuppressive treatment, incidence of comorbidities, amount of intraoperative bleeding, and surgery duration were prospectively collected.

### Surgical technique

All patients enrolled in the study had previously undergone a resection procedure for colorectal cancer with the simultaneous formation of a diverting loop ileostomy 20–30 cm proximally from the ileocecal valve at the ileal loop, which was delivered through a circular incision on the right lower abdominal wall without mesenteric torsion. Ileostomy closure, as a second operation, was performed approximately 6 months after initial surgery, after adjuvant chemotherapy (if necessary). Patients with American Joint Committee on Cancer—AJCC—stage 0 or 1 had the ileostomy removal procedure performed much earlier, as early as 14 days, after the histopathological examination results were obtained. At ileostomy closure, a circumferential incision around the ileostomy was performed. Adhesions were gently detached from the abdominal wall with scissors. After small bowel mobilization, the short-segment small bowel resection (approximately 15–25 cm) was performed. Anastomosis was performed via end-to-end single polydioxanone (PDS) Plus 4–0 running suture. Wound closure was done along three layers, including the peritoneum layer, rectus abdominis fascia, and subcutaneous layer. 2–0 absorbable PDS Plus running suture was used for peritoneum and fascia layer, and 3–0 Vicryl Plus single sutures were used to close the subcutaneous layer. In the five cases of parastomal hernia (three in the NPWT group and two in the control group) it was necessary to perform hernia repair using a polypropylene mesh, as in the sub-lay method. In those patients, the silicone drain was placed in the subfascial layer and removed at 2 or 3 postoperative days (after exudation reduction to less than 30 ml/day). In the control group, the skin was closed by six to eight single non-absorbable Monosyn 3–0 loose sutures every 7–9 mm, and a sterile wound dressing was placed. In the NPWT group, the skin was closed with 4–6 single non-absorbable Monosyn 3–0 loose sutures every 1 cm. A NANOVA negative-pressure dressing was placed over the entire length of the incision. Thanks to the use of NPWT, which stabilizes wound edges, we were able to place less skin sutures without the risk of wound dehiscence. NPWT also provides the opportunity to evacuate exudate from the subcutaneous tissue in sterile conditions and prevent the formation of a seroma or hematoma. In the control group, the first dressing change was made 48 h after the operation, and thereafter dressings were changed daily until the removal of sutures on postoperative day 7. In the NPWT group, the NANOVA dressing was taken out at 72 h. Three Steri-Strips were placed between the sutures, and a standard sterile dressing was placed. The dressing was then changed every 24 h until the removal of sutures on postoperative day 7.

### Perioperative care

All patients received second-generation cephalosporin (20 mg/kg) 30 min before the incision. On postoperative day 1, patients were fully mobilized and received a standard oral liquid diet with a volume restriction of 1 l. Patients with good diet tolerance were fed with a standard hospital diet from postoperative day 2. Perioperative care of patients in this study was compliant with the enhanced recovery after surgery (ERAS) protocol in colorectal surgery [18].

Healing was evaluated during dressing changes in the ward and then during routine check-ups in the outpatient clinic on postoperative days 7 and 14. Patients who noticed any abnormalities related to wound healing contacted the outpatient clinic by telephone and were admitted for an additional visit. After 30 days, the patients were contacted by telephone to obtain information about possible abnormalities related to healing and were asked to send a photo of the healed wound by e-mail. ForWHC, the frequency of monitoring visits was based on clinical status. The last visit to the outpatient clinic was made approximately 2 weeks after CWH.

### End point criteria

The primary endpoint was the reduction of WHC after protective ileostomy closure. WHC were defined as any condition of the wound that required postoperative intervention other than a change of dressing or removal of stiches.

Secondary endpoints were the incidence of SSI, postoperative length of hospital stay (LOS) and the duration of CWH. CWH was defined as complete closure of the wound without any secretion from the wound, as assessed at the outpatient clinic or reported by the patient. Incisional SSI diagnosis were made according to the criteria of the Center for Disease Control (CDC) and European Centre for Disease Prevention and Control (ECDC) for diagnosis of SSI [19, 20].

### Statistical analysis

Continuous data are presented as the medians and inter-quartile ranges, unless otherwise indicated. Continuous variables were compared using the Mann–Whitney test and Student’s *t*-test. Categorical variables were compared using the chi-square test, including Yates’ correction or Fisher’s exact test when necessary. The level of significance was set at *p* < 0.05. Logistic regression models were used to detect possible risk factors for WHC and SSI incidence. In the case of LOS and CWH, simple linear regressions were used to determine potentially relevant factors, and then multiple regression models were created. Analyses were performed with Statistica 13.5.

## Results

**Fig. 1:**
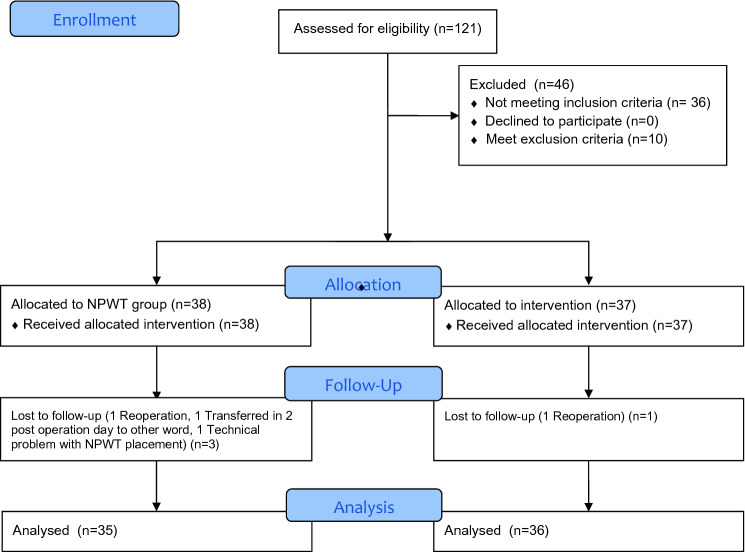
CONSORT flow diagram. *NPWT* negative-pressure wound therapy

A total of 75 patients were randomized to the study. Four patients (5.3%) were lost to follow-up (two were lost as a result of reoperation, one was transferred to another ward, and one was excluded because of a technical problem with NPWT device—difficulties with maintaining airtightness), and none of those patients developed WHC or SSI within 30 days. Patient flow through the study is presented in Fig. 1.

Table 1 shows patients’ baseline characteristics before ileostomy closure. In the two study groups, 35 patients were treated with postoperative NPWT (24 males [68.6%]; mean age 61.6 ± 11.3 years), and 36 patients (20 males[55.6%]; mean age 62.4 ± 11.3 years) were treated with suturing of the wound and traditional dressings (control group). No significant differences between the two groups were observed in patient characteristics or preoperative treatments. Surgical outcomes are shown in Table 2, and WHC are shown in Table 3. WHC were observed in 3 (8.6%) patients in the NPWT group and 11 (30.6%) in the control group (*p* = 0.020). SSI was observed in two (5.7%) patients with NPWT and in eight (22.2%) patients in the control group (*p* = 0.046). The median LOS was 3 (2–4) days in the NPWT group and did not significantly differ from that in the control group, 4 (2.5–5) days. The median duration of wound healing in patients with NPWT and in control groups was 7 (7–7) and 7 (7–15.5) days, respectively (*p* = 0.030).

**Table 1:** Groups characteristics

Parameter	Group 1 NPWT	Group 2 control	*p* value
Number of patients, *n*	35	36	n/a
Females, *n* (%)	11 (31.4%)	16 (44.4%)	0.259
Males, *n* (%)	24 (68.6%)	20 (55.6%)	
Mean age, years ± SD	61.6 ± 11.3	62.4 ± 11.3	0.974
Body mass index, kg/m^2^ ± SD	26.2 ± 4.5	26.2 ± 4.3	0.794
ASA 1, *n* (%)	1 (2.9%)	1 (2.8%)	0.943
ASA 2, *n* (%)	22 (62.9%)	24 (66.7%)	
ASA 3, *n* (%)	12 (34.2%)	11 (30.5%)	
Any comorbidity, *n* (%)	25 (71.4%)	26 (72.2%)	0.942
Cardiovascular disease, *n* (%)	11(31.4%)	7 (19.4%)	0.252
Hypertension, *n* (%)	18(51.4%)	13 (36.1%)	0.199
Diabetes, *n* (%)	5 (14.3%)	5 (13.9%)	0.962
Pulmonary disease, *n* (%)	3 (8.6%)	4 (11.1%)	0.724
Renal disease, *n* (%)	2 (5.7%)	2 (5.6%)	0.977
Other comorbidity, *n* (%)	15 (42.8%)	14 (38.9%)	0.738
Smoking, *n* (%)	5 (14.3%)	6 (16.7%)	0.785
Immunosuppressive treatment, *n* (%)	3 (8.6%)	1 (2.8%)	0.297
Radiotherapy *n* (%)	27 (77.1%)	29 (80.6%)	0.729
Chemotherapy (pre- or postoperative), *n* (%)	27 (77.1%)	28 (77.8%)	0.950
AJCC Stage 0, *n* (%)	7 (20%)	11 (30.6%)	0.319
AJCC Stage I, *n* (%)	7 (20.0%)	9 (25.0%)	
AJCC Stage II, *n* (%)	7 (20.0%)	7 (19.4%)	
AJCC Stage III, *n* (%)	13 (37.1%)	6 (16.7%)	
AJCC Stage IV, *n* (%)	1 (2.9%)	3 (8.3%)	
Median time between operations, days (IQR)	148 (17–687)	139 (25–517)	0.296
Previous operation			
Hemicolectomy, *n* (%)	4 (11.4%)	4 (11.1%)	0.829
Colectomy, *n* (%)	2 (5.7%)	2 (5.5%)	
Anterior resection of rectum *n* (%)	8 (22.9%)	13 (36.1%)	
Intersphincter resection *n* (%)	3 (8.6%)	4 (11.1%)	
TaTME, *n* (%)	18 (51.4%)	15 (41.7%)	

*NPWT* negative pressure wound therapy

*SD* standard deviation

*ASA* American Society of Anaesthesiologists class

*AJCC* American Joint Committee on Cancer tumor stage

*IQR* inter−quartile range

*TaTME* transanal total mesorectum excision

**Table 2:** Peri- and postoperative outcomes in analysed groups

Parameter	Group 1 NPWT	Group 2 control	*p* value
Number of patients, *n*	35	36	n/a
Median operative time, minutes (IQR)	57 (50–73)	55 (52–70)	0.856
Mean perioperative blood loss, ml ± SD	16 ± 9	18 ± 10	0.654
Patients without any complications, *n* (%)	30 (85.7%)	23 (63.9%)	**0.035**
Patients with complications, *n* (%)	5 (14.3%)	13 (36.1%)	
Clavien-Dindo grade 1, *n* (%)	4 (11.4%)	6 (16.7%)	n/a
Clavien-Dindo grade 2, *n* (%)	1 (2.9%)	5 (13.9%)	
Clavien-Dindo grade 3, *n* (%)	–	2 (5.6%)	
Clavien-Dindo grade 4, *n* (%)	–	–	
Clavien-Dindo grade 5, *n* (%)	–	–	
Median length of postoperative hospital stay, days (IQR)	3 (2–4)	4 (2.5–5)	0.072
Median duration of complete wound healing time days (IQR)	7 (7–7)	7 (7–15.5)	**0.030**

Significant *p*-values were marked with bolded font

*NPWT* negative pressure wound therapy

*SD* standard deviation

*IQR* inter−quartile range

**Table 3:** Postoperative wound management complications (WMC)

Complication	Group 1 NPWT	Group 2 Control	*p* value
Number of WMC, *n* (%)	3 (8.57%)	11(30.6%)	**0.020**
Surgical site infections (SSI), *n* (%)	2 (5.71%)	8 (22.2%)	**0.046**
Superficial SSI, *n* (%)	2 (5.71%)	4 (11.1%)	n/a
Deep SSI, *n* (%)	0	3 (38.8%)	n/a
Organ SSI 1, *n* (%)	0	1 (2.8%)	n/a
Hematoma, *n* (%)	0	3 (8.3%)	n/a
Seroma, *n* (%)	1 (2.9%)	1(2.8%)	0.984

Significant *p*-values were marked with bolded font

To identify potential risk factors for WHC, univariate logistic regression models were constructed, as presented in Table 4. The univariate analyses revealed that only postoperative NPWT significantly decreased the odds ratio for WHC incidence. In the case of risk factors for SSI, none of the factors analyzed in the univariate regression model were statistically significant. Simple linear regression models were built for the length of hospital stay and CWH time. In the LOS regression model, the adjusted *R*^2^ for multiple regression was 60.68% with a *p* value < 0.001. In this model, the factors identified as significantly prolonging LOS were: SSI by 4.58 days (2.62–6.54), other complications by 8.65 days (6.20–11.10), and BMI < 18.5 kg/m^2^ by 7.36 days (1.22–13.50). Of all the factors analyzed in the simple regression models, only postoperative use of NPWT was significant. NPWT use was associated with shortening CWH by 3.00 ± 1.24 days in simple regression model with an adjusted *R*^2^ of 6.42% (*p* value < 0.001).

**Table 4:** Univariate logistic regression analyses of WMC incidence

Parameter	OR (95% CI)	*p* value
Postoperative NPWT (yes vs. no)	0.19 (0.04–0.97)	**0.041**
Sex (female vs. male)	0.74 (0.15–3.72)	0.704
Age (≥ 75 vs. < 75 years)	1.72 (0.16–18.91)	0.650
BMI (≥ 30 vs. < 30 kg/m^2^)	2.07 (0.33–13.18)	0.429
Smoking (yes vs. no)	0.34 (0.03–4.68)	0.719
Radiotherapy (yes vs. no)	1.02 (0.04–27.13)	0.992
Chemotherapy (yes vs. no)	0.92 (0.04–23.07)	0.926
Paraostomal hernioplasty (yes vs. no)	0.44 (0.03–5.98)	0.532
Time between previous operation (≥ 365 vs < 365 days)	3.08 (0.23–40.65)	0.381
Cardiovascular disease (yes vs. no)	0.67 (0.10–4.69)	0.685
Diabetes (yes vs. no)	1.44 (0.19–11.13)	0.719
Pulmonary disease (yes vs. no)	0.79 (0.06–10.61)	0.858
Renal disease (yes vs. no)	3.39 (0.13–90.93)	0.457
TaTME (yes vs. no)	1.25 (0.25–6.34)	0.780
AJCC IV (yes vs. no)	2.19 (0.09–51.96)	0.619
Complications not related to wound management	0.83 (0.25–2.77)	0.762

Significant *p*-value was marked with bolded font

*WMC* wound management complication

*NPWT* negative pressure wound therapy

*BMI* body mass index

*TaTME* transanal total mesorectum excision

*AJCC* American Joint Committee on Cancer tumor stage

## Discussion

To the best of our knowledge, this is the first study confirming the usefulness of postoperative NPWT in reducing the number of WHC associated with elective ileostomy closure in patients after surgery for colorectal cancer [14, 15]. Our study showed that the use of postoperative NPWT after ileostomy reversal procedures significantly reduces the risk of complications. This may have significant clinical implications, especially taking into considerations studies that suggest the benefits of early closure of the ileostomy, even before adjuvant chemotherapy [7]. However, concern over complications after ileostomy closure often postpones this procedure until the end of adjuvant chemotherapy. Postoperative NPWT may be an adequate solution of that clinical problem.

Although the risk of anastomotic leak after colorectal cancer surgery is reduced by diverting ileostomy, opponents argue it creates the need for another surgery with a relatively high risk of complications [21]. Although those tend to be relatively mild and local complications, they may cause a delay in adjuvant chemotherapy, which diminishes the results obtained by oncological treatment. One method to minimize the risk of complications after the ileostomy closure is protective NPWT placement. There is a very large divergence in the reported incidences of infectious complications after ileostomy reversal. Studies that deal with other issues related to surgery report a very low incidence of these complications [21]; however, research focusing on the impact of various surgical techniques on the incidence of SSI reports up to 40% risk of SSI in control groups. This relatively high incidence of SSI has led the American College of Surgeons (ACS) to recommend closing wounds after ileostomy reversal using the purse-string technique [10]. Our study was designed between 2015 and 2016, prior to the 2017 publication of the ACS recommendations to use the purse-string technique for closure of this type of wound [10]. Until then, simple suture technique was the standard, which is why in the control group simple suturing of the wound was applied. Because the proposed technique significantly extends healing time and often, especially in obese people, gives an unsatisfactory cosmetic effect [9, 11, 12], many surgeons still close their ileostomy wounds with conventional primary closure [21]. The use of postoperative NPWT provides the opportunity to combine the benefits of both techniques. We suppose, based on our experience, that the healing time and final cosmetic effect do not differ from those of the primary closure technique. Additionally, NPWT offers a significant reduction in the occurrence of WMC and SSI compared with the effects of using the purse-string technique [11–13].

Several clinical trials have investigated the usefulness of postoperative NPWT in reducing postoperative infections, but the majority of them have been conducted in fields other than gastrointestinal surgery [15]. Only a few studies have been conducted in the field of general, oncological, or digestive surgery [14], and only one article has described the use of NPWT as a postoperative dressing in patients after diverting ileostomy closure [22]. The results of this study differ from our observations. In the cited study, the wound after ileostomy was closed using the purse-string suture technique; for this reason, even in the group without complications, the wound healing time was longer than 30 days [22]. In our study, even in the group of patients with an infectious complication, CWH was shorter than 30 days. In our research, the NPWT application was limited to 72 h after the surgery and was used only to evacuate the exudate or hematoma from the wound. The dressing was placed in sterile conditions of the operating room over the cleaned wound while the antibiotic prophylaxis used for the operation was still in effect. In the cited study, a NPWT was installed 24 h after surgery and maintained for more than 2 weeks, which in our opinion may increase the potential for colonization of the wound and possible development of infectious complications. In the previously cited meta-analysis of all other clinical trials of NPWT in the postoperative period, its use was shorter, ranging from 3 to 7 days, and the dressing was placed over the wound immediately after the surgery [15]. Another difference between our study that of Uchino et al. concerns the intervention population. Our research included patients who underwent surgery for colorectal cancer, mainly rectal cancer, which particularly exposes them to the risk of infectious complications. The majority of them were elderly with numerous comorbidities and higher BMI. Additionally, most of them underwent radio- and chemotherapy shortly before the surgery. In the Uchino study, the population consisted of patients who had surgery as a result of ulcerative colitis, resulting in a significantly lower number of infectious complications in the control group than in our study. Given these differences, we believe that these two studies are not directly comparable, as they concern different issues. To increase the chance of achieving statistical significance while limiting the necessary study population, the cumulative index of WHC, not the occurrence of SSI, was established as the primary outcome. With the incidence of SSI in our group of patients and expected reduction of SSI cases by approximately 50%, the minimal sample size would have required recruitment of 80 patients in each arm. However, the observed effect of intervention exceeded our expectations. A statistically significant effect of the use of NPWT on reducing the incidence of SSI was confirmed.

Our study showed that the use of postoperative NPWT dressing is safe. In the NPWT group, there was no increase in the percentage of postoperative complications, as well as no case of a complication that could be linked directly to NPWT use (postoperative bleeding or entero-cutaneous fistula formation). In the group of patients with NPWT, both the time of hospitalization and the time of healing of the surgical wound were shortened.

### Limitations

Because of the actual incidence of WHC in this study differing from the values assumed during the sample size calculation, the post hoc analysis revealed that the study of the primary outcome, despite obtaining statistically significant differences, achieved 65% power and not the assumed 80%. The obtained results are therefore underpowered.

Also, our study used only one type of NPWT device. Comparison of the effectiveness and safety of other types of NPWT equipment in this application will require further research.

We did not specifically analyze the time period between chemotherapy and operation, but we observed no difference in the incidence of postoperative chemotherapy between groups, and the time from colorectal resection and ileostomy closure until chemotherapy did not differ between groups.

We did not perform an analysis of the cosmetic effect of our treatment. Further research in this area is required.

Lastly, this study was a single-center study, which, on one hand, is a strength in the consistent treatment of all patients, but on the other hand, it will require confirmation in multi-center studies on larger groups of patients.

## Conclusions

Prophylactic postoperative NPWT after diverting ileostomy closure in colorectal cancer patients reduces the incidence of WHC, SSI, and complete wound healing time.
